# Lipid-based nanoparticles as drug delivery carriers for cancer therapy

**DOI:** 10.3389/fonc.2024.1296091

**Published:** 2024-04-10

**Authors:** Ibtesam Waheed, Anwar Ali, Huma Tabassum, Narjis Khatoon, Wing-Fu Lai, Xin Zhou

**Affiliations:** ^1^ Institute of Comparative Medicine, College of Veterinary Medicine, Yangzhou University, Yangzhou, China; ^2^ Department of Applied Biology and Chemical Technology, Hong Kong Polytechnic University, Kowloon, Hong Kong SAR, China; ^3^ Department of Biochemical and Biotechnological Sciences, School of Precision Medicine, University of Campania, Naples, Italy; ^4^ Institute of Social and Cultural Studies, Department of Public Health, University of the Punjab, Lahore, Pakistan; ^5^ Department of Biotechnology, Lahore College for Women University, Lahore, Pakistan; ^6^ School of Food Science and Nutrition, University of Leeds, Leeds, United Kingdom

**Keywords:** lipid-based nanoparticles, nanomedicine, drug delivery, active targeting, passive targeting, cancer therapy

## Abstract

Cancer is a severe disease that results in death in all countries of the world. A nano-based drug delivery approach is the best alternative, directly targeting cancer tumor cells with improved drug cellular uptake. Different types of nanoparticle-based drug carriers are advanced for the treatment of cancer, and to increase the therapeutic effectiveness and safety of cancer therapy, many substances have been looked into as drug carriers. Lipid-based nanoparticles (LBNPs) have significantly attracted interest recently. These natural biomolecules that alternate to other polymers are frequently recycled in medicine due to their amphipathic properties. Lipid nanoparticles typically provide a variety of benefits, including biocompatibility and biodegradability. This review covers different classes of LBNPs, including their characterization and different synthesis technologies. This review discusses the most significant advancements in lipid nanoparticle technology and their use in medicine administration. Moreover, the review also emphasized the applications of lipid nanoparticles that are used in different cancer treatment types.

## 1 Introduction

Cancer continues to be an increasingly widespread disease, and it is expected that cancer will be the second most dangerous disease after heart, which may lead to death (1). According to the World Health Organization, there will be 15 million new instances of cancer by 2024 (1). Drug delivery via nanoparticles has been extensively studied for decades (2). It has been clinically proven that lipid-based nanoparticles, including liposomes, solid lipid nanoparticles (SLNs), and nanostructured lipid carriers (NLCs) are incredibly effective at delivering both hydrophobic and hydrophilic medicines (2, 3). Doxil is a polyethylene glycol (PEG)ylated liposome containing doxorubicin (DOX), the first FDA-approved nanodrug to treat solid tumors such as breast and ovarian cancer (4). PEGylated liposomal doxorubicin Doxil provides several benefits over free DOX (4). These include a significantly lower risk of cardiotoxicity, a longer plasma retention time, and passive tumor targeting thanks to the enhanced permeability and retention (EPR) effect. A significant turning point for lipid-based drug delivery systems and cancer nanomedicine was the clinical approval of Doxil in 1995 (5).

The efficacy of lipid-based nanoparticles (LBNPs) largely depends on the enhanced permeability and retention (EPR) effect, especially when it comes to passive tumor targeting. The aberrant vasculature and compromised lymphatic outflow frequently seen in tumor tissues are referred to as the “EPR effect”. It is common for tumors to have leaky blood arteries with irregular fenestrations, which makes it possible for passive extravasation of bloodstream nanoparticles into the tumor interstitial (6). Concurrently, the tumors’ impaired lymphatic outflow makes it more difficult for these nanoparticles to be cleared effectively. This special mixture minimizes the dissemination of nanoparticles in healthy tissues while causing a selective concentration within the malignant tissue (7). To deliver drugs to tumors specifically, the EPR effect, which is tailored to lipid-based nanoparticles, is crucial for LBNPs. Therapeutic agent concentration in the tumor microenvironment is increased via passive targeting by the EPR effect. This selective accumulation reduces systemic side effects by increasing drug delivery to cancer cells while minimizing exposure to normal tissues, which is very useful in treating cancer (8).

However, because of their exceptional biocompatibility, biodegradability, and entrapment effectiveness, LBNPs have also been acknowledged as an appropriate carrier for nucleic acids such as DNA, mRNA, and siRNA (9). The exponential growth of publications since the 1990s proves the LBNPs’ ongoing success in treating various diseases and demonstrating their immense promise as next-generation drug delivery vehicles. LBNPs can be divided into three systems based on their nanostructure: liposomes, SLNs, and NLCs (10). The first approved double-stranded small interfering RNA in 2018 delivering LBNP is called ONPATTRO (patisiran) (11). In fact, since the 1980s, LBNPs with cationic lipids or pH-responsive lipids have been used to encapsulate and distribute nucleic acids (12). Ionizable cationic lipids are advantageous when creating LBNP systems since they have positive charges at lower pH values (pH < 6.0) but are neutral at physiological pH. LNPs made of cholesterol, phospholipid (1,2-distearoyl-sn-glycero-3-phosphocholine [DSPC]), ionizable cationic lipids (DLin-MC3-DMA), and polyethylene glycol-modified lipids (PEG2000-C-DMG) ensnape siRNA.

The PEG2000-C-DMG coating is replaced during systemic circulation by apolipoprotein E (Apo E), which is drawn to the liver by cholesterol and then endocytosed by hepatocytes (13). The acidic endosome state disrupts the endosomal membranes, causing DLin-MC3-DMA in the LBNPs to become positively charged upon entering the endosome and releasing the RNA cargo into the cytoplasm to fulfill its role. This review includes the development and innovation of lipid-based nanoparticles that have been adopted and research for cancer treatment. First, different LBNP types, characterizations, and properties were discussed. Subsequently, various techniques for producing these LBNPs, including solvent-based emulsification, nonsolvent emulsification, bulk nanoprecipitation, microfluidic approaches, and coacervation technology, were elaborated. In conclusion, we discussed LBNPs and their potential effects on different cancer types.

## 2 Characterization of LBNPs

### 2.1 Morphology of lipid-based nanoparticles

The most common nanoparticles that are primarily employed for research have a spherical shape, according to the synthesis process of nanoparticle formation (14). These spherical nanoparticles are formed using a sol-gel approach and the electrostatic interaction between the polar or nonpolar bond in the solution helps to keep spherical morphology (14). Polymerization contributes to the destruction of spherical liposomes into random-shaped particles. The morphology of spherical liposomes might change due to the polymerization process, creating irregularly shaped particles. The structural integrity of the liposomal membrane may be compromised during the polymerization process of liposomes due to related chemical and physical alterations (15). The initially spherical liposomes are frequently deformed into erratic and less recognizable shapes due to this disturbance. Polymerization substantially affects liposome morphology since the shape change affects important characteristics like stability, surface area, and drug encapsulation effectiveness. Designing and regulating polymerization’s impact on liposomal morphology is essential to maximizing the performance of drug delivery systems based on liposomes (16).

Additionally, platelets may result from changes to the content of lipid mixtures and lipid forms rather than spherical particles in the preparation of SLNs and NLCs (17). In addition, chemical composition and process parameters such as pH and temperature significantly influence the shape of the generated nanoparticles (18).

### 2.2 Shape and size distribution

The produced particle’s morphological feathers (size and shape) are essential aspects when considering a drug delivery carrier (19). The smaller-sized particles have more surface area and drug-loading capability. In addition to this, the smaller nanosized particles can be easily removed during urine or capillary lungs and vice versa (20). In another study, lipid-based drug delivery systems with a hydrodynamic diameter of 5.5 nm were used as drug carriers, and it was observed that these particles are easily removed through urinary excretion (21). It has been demonstrated that size directly influences how well lipid-based nanoparticles like liposomes are absorbed in the spleen (22). The relationship between hepatic absorption and particle size for lipid particles in the liver is less clear (23). For instance, it was discovered that the endothelium sinusoidal cells in a healthy human liver exhibit fenestrae with pores measuring 6.5 nm in diameter. The liver was taken more readily than liposome, which is 165.5–275.0 nm in size (136.2 nm and 318.0.0 nm) (24). In this analysis, the liposomes were largely absorbed by the liver. Interestingly, various species’ endothelial fenestra diameters fluctuate, and this feature is equally crucial concerning the variances in nanoparticle sizes that have been observed to enter the liver parenchyma (25).

As a result, the nanoparticle’s size is a central element that must be carefully considered, as it determines the biodistribution of the drug supply system for nanoparticles (26). The pores are the significant sites of nanoparticles that accumulate information. It is also important to consider other factors, such as surface stress and particle shape, which make it challenging to prepare a final nanoprogram. It must be remembered that liposomes can also contribute to squeezing large particles through narrow, intercellular pores (27). An optimal colloidal size must typically be between 100 nm and 300 nm to ensure enough circulation duration and tumor growth, based on the exterior charge and other factors.

### 2.3 Surface charge

Nanoparticles have a great volume-to-surface relationship compared to higher elements, so it is important to assess and monitor their surface characteristics accurately (28). A ZP analyzer can determine the surface charge, also known as zeta potential (ZP). The degree of repulsion force can be utilized to indicate the stability of colloidal dispersions that have been prepared. Particles are prevented from aggregating by a strong repulsion force. Generally, ZP values greater than +30 mV or less than −30 mV are regarded as powerful enough to oppose one another and maintain electrostatic equilibrium. Noteworthy is the fact that LBNP formulations with polyhydroxy and other nonionic surfactants tend to possess reduced ZP levels. Meanwhile, reports have stated that the ZP value of LNPs increases with an increase in oil content. However, a near-neutral charge is preferred for drug delivery that is systematic. However, a near-neutral charge is preferred for drug administration that is systematic. Therefore, screening out strong charges using PEGylation or another surface modification, like coating the LBNPs with nonionic surfactants like Tween 80, is necessary.

Studies have repeatedly shown that the stability of colloidal dispersions is influenced by surface charge, as shown by the zeta potential. A study by Pochapski et al. (29) examined the impact of surface charge on liposome stability. By changing the lipid bilayer’s composition, the researchers could vary the zeta potential of the liposomes. When compared to liposomes with a near-neutral zeta potential, they discovered that liposomes with a higher positive or negative zeta potential showed better stability (30). This showed that preventing aggregation and improving colloidal stability mainly depend on electrostatic repulsion between charged particles. Similarly, Zielińska et al. (31) investigated how polymeric nanoparticle stability was affected by zeta potential. They found a strong relationship between the stability of the colloidal dispersion and the zeta potential by adding charged polymers to change the surface charge of nanoparticles (32). Higher zeta potential nanoparticles—positive or negative—showed improved stability because of stronger electrostatic repulsion, which prevented the particles from aggregating.

### 2.4 Phase transition temperature

Liposomes experience variations in viscosity, can deliver drugs, and interact with reticuloendothelial system (RES) as temperature changes, in contrast to polymeric nanoparticles, which are generally temperature-insensitive (33). This is crucial for the administration of liposomal medications because lipophilic compounds are incorporated into the hydrophobic nucleus of the phospholipid bilayer, while hydrophilic molecules are enclosed inside the liposomal aqueous interior, encompassed by the phospholipid bilayer (34). Various lipids have distinct phase transition temperatures (PTT) and live above or below this temperature in different physical conditions. When temperatures are below the PTT, lipids normally have a more structured and well-ordered orientation (solid, gel-like phase), and when temperatures are above the PTT, they usually have a liquid-crystalline (fluid) phase (35). Adding various cholesterol concentrations can lessen the impact of PTT on liposomes (36). When high cholesterol concentrations are added at temperatures above their PTT, the liposomal bilayer becomes less elastic and leaky, thus making the liposomes more stable.

### 2.5 Plasma protein interaction particle stability and clearance

When delivered into the circulation, plasma proteins, including lipoproteins and complementary proteins, may be affected by nanoparticle interactions (37). This interaction is crucial because it affects how the system responds to nanoparticles and how stable its clearance is. More specifically, lipid-based nanoparticles are more susceptible to protein stability than other nanoparticles because of their interactions with certain proteins that might result in particle deformation and the leaking of encapsulated content (38). Other plasma proteins, including immunoglobulins and albumin, have been shown to also affect liposome clearance and stability. For example, it has been studied that IgG antibodies of rabbits covalently attached to liposomes increase the liposome absorption by rat buffer cells (39). Furthermore, the liposome action level was recorded when incubated in bovine serum albumin.

Additionally, the small quantity of lipoprotein contaminating commercial albumin formulations may have contributed to the destabilizing effect (40). Recently, it has been shown that albumin and IgG can infiltrate the liposomal bilayer. However, there was no liposome rupture during the encounter (41).

## 3 Classification of LBNPs

**Figure 1:**
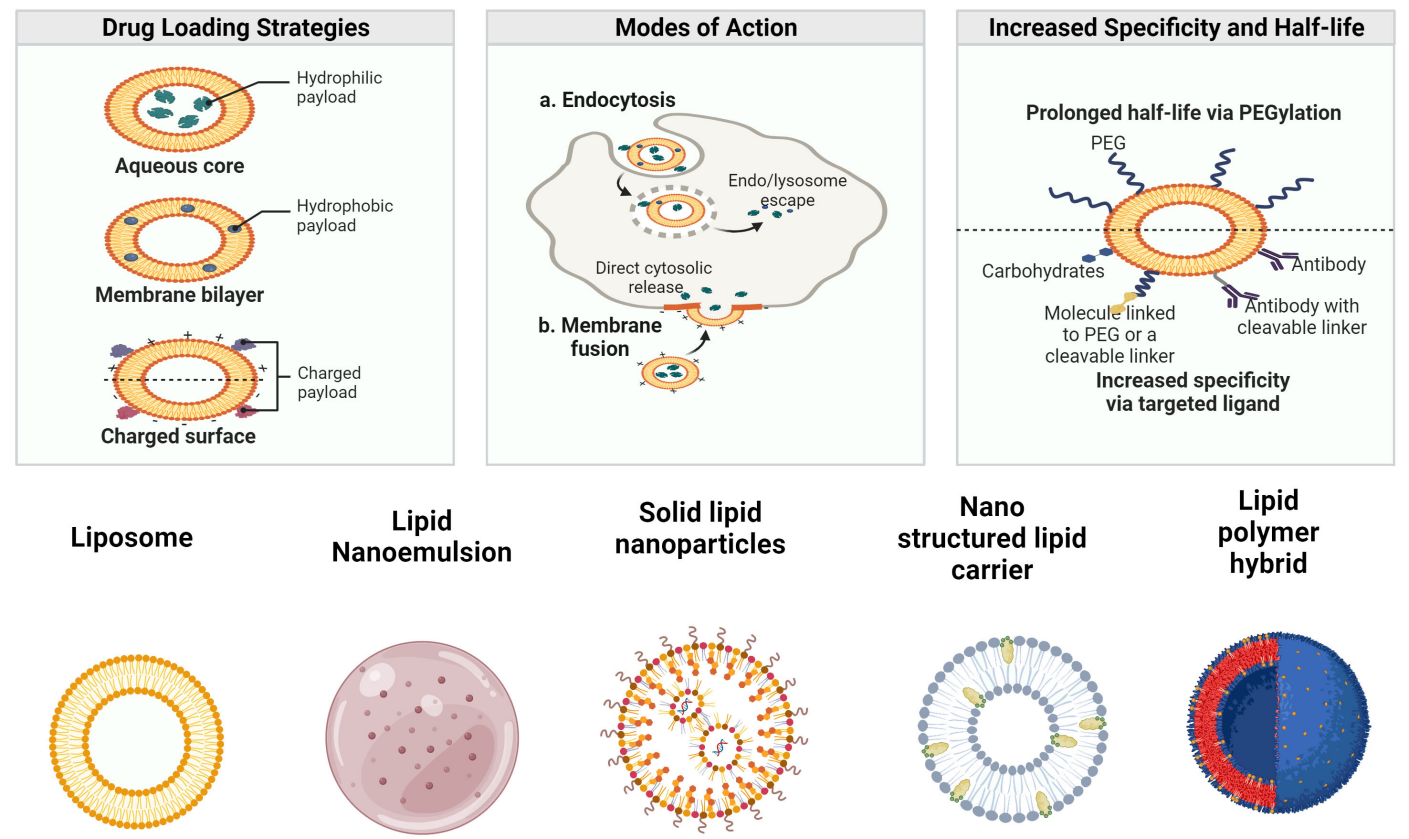
Drug delivery of LBNPs and their types.

To manage the quality of LBNPs and ensure that they meet the requirements of diverse applications, proper characterization of LBNPs is essential (42). Particle size and polydispersity index (PDI), ZP, surface shape, encapsulation efficiency (EE), drug release, crystallinity, and nanoparticle stability are crucial LBNP features that must be thoroughly characterized (43). As a bioactive delivery system, LBNPs have more advantages. Some of the drug delivery characteristics of LBNPs and their classifications are shown in **Figure 1**. These carriers are typically investigated to enhance oral biological availability to support drug release (44). LBNPs provide several benefits, such as easy formulation, self-assembly, biocompatibility, high bioavailability, capacity for large payloads, and various physicochemical features that can be tuned to influence biological parameters (45). LBNPs are the most prevalent class of nanomedicines that have received FDA approval due to these factors (**Table 1**).

**Table 1:** Nanomedicine and their applications.

Nanoparticles	Drug	Method	Lipid	Disease counter	Surfactant	References
Mannosylated LBNPs	Doxorubicin	Injection of solvent	Tristearin, stearyl amine	Lung cancer	Soya lecithin	Shinde and Lala (46)
Lactoferrin-modified LBNPs	Docetaxel	Solvent evaporation and emulsification	Stearic acid	Tumor of brain	Soy lecithin	Elumalai et al. (47)
LBNPs	Camptothecin	Nonsolvent emulsification	Wx cetyl palmitate	Glioblastoma	Polysorbate 60 or 80	Zhong et al. (48)
LBNPs	Curcumin	Emulsion solvent evaporation	Stearic acid	Asthma	Myrj52	
LBNPs	Isoniazid	Nonsolvent emulsification	Compritol 888 ATO	Tuberculosis	Soy lecithin	
pH-responsive LBNPs	Doxorubicin	Microemulsion evaporation	Sodium laurate	Tumor	PEG	Shinn et al. (49)
Chitosan-coated LBNPs	Rifampicin	Microemulsion	Cetyl palmitate	Tuberculosis	Tween 80	Vieira et al. (50)
Magnetic LBNPs	Sorafenib	Microemulsion solvent evaporation	Cetyl palmitate	Lung cancer	Tween 80	Luiz et al. (51)

### 3.1 Liposomes

Liposomes are recognized as one of the most important delivery mechanisms for their biocompatibility and biodegradability (52). Phospholipids are the main important element of these nanoparticles, having amphipathic properties arranged in a bilayer structure (53). When incorporated into their structure, they form vesicles when there is water present, which improves the stability and solubility of cancer therapies (54). They contain either hydrophobic or hydrophilic medicinal products. Other substances, such as cholesterol, phospholipids, and other ingredients, may be added into their preparations to reduce nanoparticle fluidity and increase hydrophobic drug permeability via the bilayer membrane, improving the stability of these nanoparticles in the blood (55). Lately, there has been an exhaustive exploration of the blend and creation of new liposomes. In reality, Fe_3_O_4_ cores are progressively being employed to functionalize many types of nanoparticles (56). In 2014, liposome-encapsulated DOX with citric acid-coated magnetic nanoparticles was used to manage chemotherapy and hyperthermia (57).

In addition, ultrasound-sensitive liposomes were established for doxorubicin DOX encapsulation, as in the scenario of thermosensitive polymer (NIPMAM-co-NIPAM) that can be deteriorated by sonication, resulting in coencapsulation of drug released in 2014 (58). Doxorubicin was coencapsulated with Magnevist^®^, a contrast agent. Both active ingredients were included in a liposome altered with amphiphilic properties (59). When used to treat ovarian cancer, this novel liposome-based doxorubicin (DOX) formulation has shown to be both safe and effective. Two distinct anticancer drugs, PTX and resveratrol, can be combined in a 50-nm-sized delivery system using PEGylated liposomes. Investigation has proven that both *in vivo* and *in vitro* liposome therapies have been more effective than other agent therapies (60). Different liposomes for encapsulating various anticancer drugs were developed in 2018 (61). For example, a liposome that has been modified to use 5-fluorouracil (5-FU) for cancer treatment through penetrating peptides and transfer (62).

### 3.2 Solid lipid nanoparticles

SLNs, a novel method of administering colloidal drugs, consist of physiological fluids that are solid at room temperature as well as body temperature (63). These particles range in size from 50 nm to 1,000 nm. The solid lipid used is a drug-encapsulating matrix material of complex glyceride combinations and fatty acids. A combination of surfactants or polymers stabilized this matrix (64). SLNs are significantly advantageous, with characteristics such as long-term physical stability, site-specific targeting, and the potential for both hydrophilic and lipophilic medicines to have regulated release, labile pharmaceutical safety, low cost, readiness, and nontoxicity (65). Furthermore, SLNs caused very little toxicity to human granulocytes. Because of all these advantages, they are a significant candidate for pharmaceutical administration systems. The benefits of SLNs include targeting drugs and monitoring their release (66). As SLNs are composed of biocompatible and physiological lipids, they are less toxic than polymeric nanoparticles (67). They work well with both hydrophilic and lipophilic drugs, preventing contact with hydrophobic solvent macromolecules (68). SLNs are useful in the delivery of macromolecules by dermal, pulmonary, oral, intravenous, and ophthalmic routes (69).

### 3.3 Nanostructured lipid carriers

NLCs, which include liquid and solid lipids in different proportions, are an example of the second generation of SLN-based lipid **n**anocarriers (70). This method has been developed to resolve the limitations of NLCs. These limitations include their ability to load medications, especially hydrophilic pharmaceuticals, because the lipid-based core is hydrophobic by nature. Maintaining consistent particle size and composition becomes increasingly difficult as production scales up (71). Ostwald ripening, drug leakage, and aggregation are stability problems that could jeopardize long-term storage. The manufacturing process is intricate, requiring specialized tools and knowledge to complete steps like high-pressure homogenization and microemulsion (72). Thorough safety evaluations are required because of **concerns** regarding the biocompatibility and possible toxicity of the lipids and surfactants utilized in NLC formulations. The intricacy is increased by knowing the biodistribution, *in vivo* fate, and regulatory approval. Furthermore, the overall cost-effectiveness of NLCs compared to traditional delivery systems may be impacted by costs related to manufacturing processes and high-quality lipids (73).

**Figure 2:**
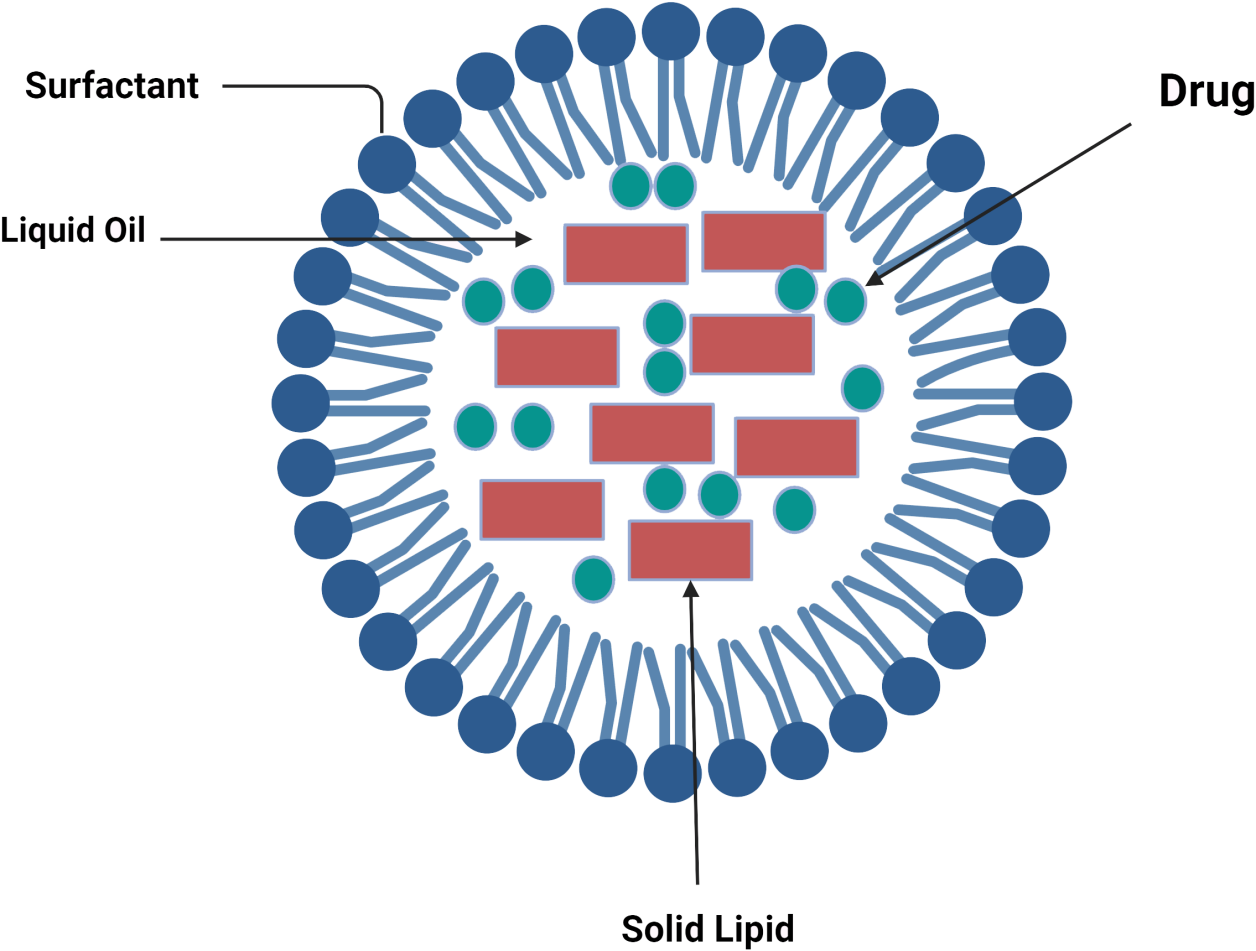
Lipid components of nanostructured lipid carriers (76).

Thus, NLCs could load more drugs and might also stop lipid crystallization brought on by fluid formulation in NLC formulation, hence avoiding medication ejection during storage (74). SLNs are composed only of solid lipids, while NLCs are a mixture of liquid and solid lipids, such as isopropyl myristate, ethyl oleate, glycerol dilates, and glycerol tricaprylate (75). The lipids’ composition and production impact the moderate particle sizes, which are in the 10–1,000-nm range and similar to SLNs (**Figure 2**).

These nanoparticles have the key advantages of being filled with hydrophilic and hydrophobic medicinal materials, being able to modify the surface by giving site-specific targeting and monitoring the medication release, and having minimal toxicity *in vivo* (77). However, there are still a few disadvantages, such as drug expulsion from the nanocarrier matrix after a polymorphic transition during storage and low load power.

## 4 LBNP synthesis

**Figure 3:**
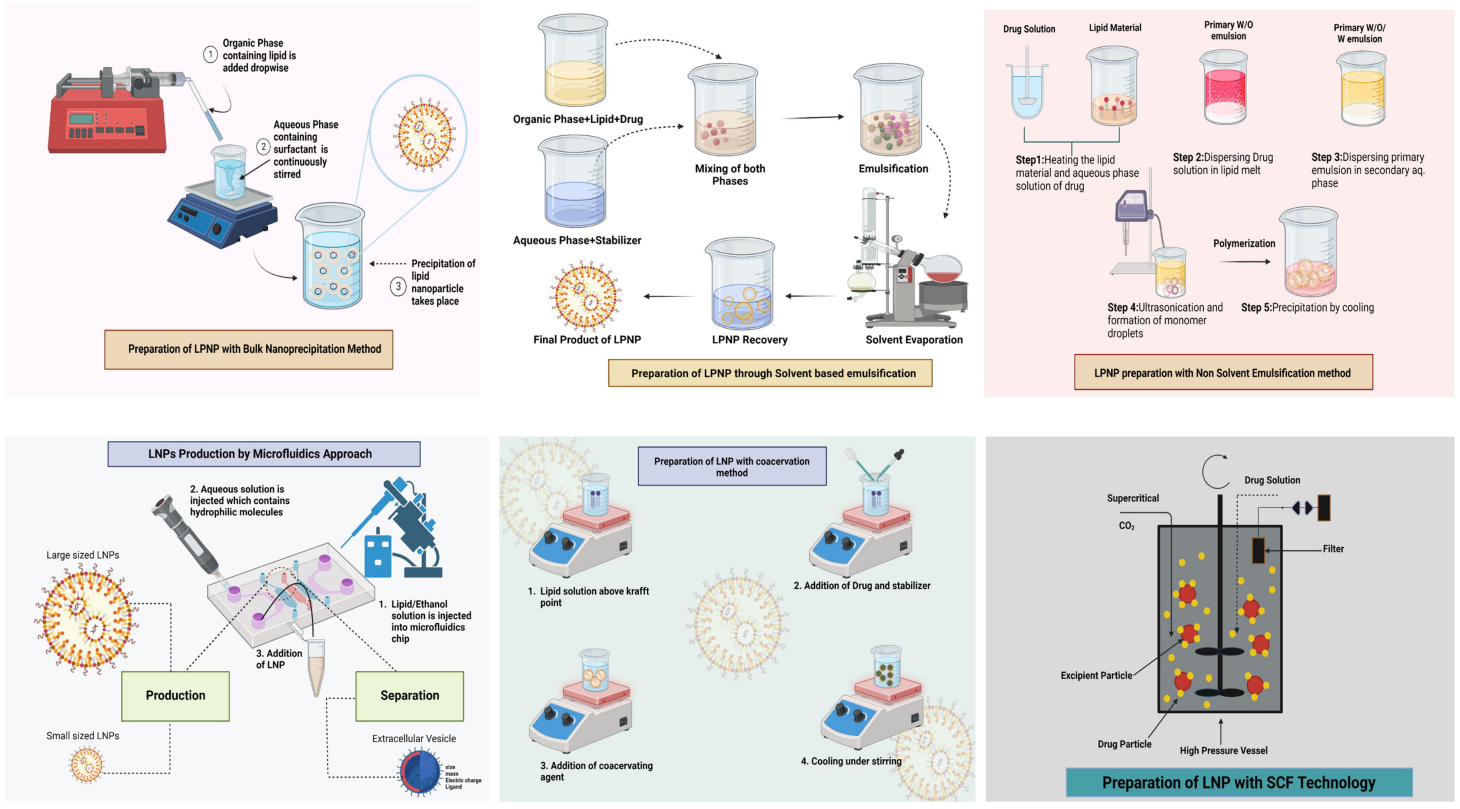
Different methods to produce LBNPs.

The creation of LBNPs has been accomplished using a wide range of techniques. Both SLNs and NLCs can be prepared using the majority of techniques. This section introduces various methods frequently used to produce LBNPs (**Figure 3**). Their benefits and drawbacks are examined, offering some criteria for choosing the best approach for a particular class of LBNPs.

### 4.1 Bulk nanoprecipitation

Fessi et al. in 1989 initially created and patented nanoprecipitation, also known as solvent displacement (78). An aqueous phase is combined with a water-miscible solvent dubbed the “organic phase” that contains lipids and hydrophobic medicines (79). Rapid precipitation of LNPs and prompt drug encapsulation result from rapid desolvation of the lipids and medicines. LBNPs are typically made by combining an organic phase with an aqueous phase, while magnetic stirring takes place in bulk solution (26). Raghuwanshi and Jena’s process of nanoprecipitation is impacted by the Marangoni effect, a complicated and cumulative phenomenon of interfacial turbulence brought on by changes in flow, diffusion, and surface tension at the interface of two miscible liquids. Amphotericin B-loaded glyceryl dilaurate-formulated nanoparticles that were easily redispersed in water and remained stable for 3 months under refrigeration were created by Chaudhari et al. via nanoprecipitation (79). For the targeted delivery of nevirapine to the brain, polysorbate 80-coated kokum butter LBNPs were created through nanoprecipitation (80). After being administered to Wistar rats, a sustained release lasting more than 24 h was noticed. Making LBNPs for gene treatments has also been done via nanoprecipitation (80). Huang et al. (81) created siRNA-encapsulated LBNPs for ethanol injection treatment of retinal disorders. By altering variables like stirring rate, organic solvent/antisolvent ratio, and lipid/surfactant/drug concentration, it is possible to tailor the size of nanoparticles and drug EE (82). A uniform and supersaturated solution of lipids is preferred for spontaneous nucleation to reduce the range of nanoparticle sizes (83). Changing the mixing time can significantly minimize nanoprecipitation. Mixing over a shorter period than the average precipitation duration might result in smaller nanoparticle sizes; mixing is completed before precipitation occurs (84). However, the primary disadvantage of bulk nanoprecipitation is the inadequate control of fluidic dynamics, which leads to nanoparticles with a larger size distribution, especially for large mixing volumes and large-scale production (79). Additionally, insufficient mixing may result in batch-to-batch variations in the quality and properties of the nanoparticles, making them unsuitable for mass manufacturing (85).

### 4.2 Method of solvent-based emulsification

The solvent-based emulsification technique, which includes solvent injection, displacement, diffusion, and emulsion-solvent evaporation, has been extensively employed to create LBNPs (86). In summary, solid lipids and hydrophobic medications are dissolved in an organic solvent that is immiscible with water to form oil-in-water emulsions (such as cyclohexane, toluene, and chloroform) (87). Thereafter, as the organic solvent evaporates, LBNPs are produced (87). The process can be used to encapsulate temperature-sensitive pharmaceuticals. However, completely removing organic solvents may be challenging, particularly if the lipids utilized are not highly soluble in the solvent, which could lead to residual solvent toxicity (79).

### 4.3 Method of nonsolvent emulsification

Nonsolvent emulsification techniques, also known as melting emulsification techniques, use melted lipids as the liquid phase to create oil-in-water emulsions rather than solvents to dissolve lipids (88). Solid lipids are often melted into liquid at 5°C to 10°C above melting temperatures (89). The melted lipids are then combined with an aqueous surfactant solution to create nanoemulsions through the use of membrane emulsification, high-pressure homogenization (HPH), microemulsions, high-speed stirring, or ultrasonication (90). It is possible to create dispersed SLNs by chilling the nanoemulsions in an ice bath (89). LBNP production and its characteristics have been influenced by several factors, including homogenization duration, sonication time, surfactant concentration, lipid concentration, drug concentration, lipid type, and surfactant type (91). The solubility of medicine in lipids was found to impact drug loading capacity among them. The ideal surfactant should have a hydrophilic–lipophilic balance (HLB) value between 12 and 16, such as Chromophore EL (12–14), Tween 20 (16), and Tween 80 (15) when choosing a surfactant (79). Additionally, a more extended sonication period and increased surfactant concentration resulted in smaller particle sizes (92). When the initial drug loading was less than 0.75%, it had no adverse effects on the size of the lipid particles, but when it exceeded 1%, the size of the particles significantly increased (79). Drug crystals were created as a result of the unencapsulated medications crystallizing when the drug loading was increased to 2% (93). Unlike solvent-based emulsification procedures, nonsolvent-based approaches reduce the potential for residual solvent toxicity in the LBNP solution by not using harmful organic solvents (94). However, drug loading capacity and EE may be constrained by a drug’s solubility in lipids. Poor drug loading occurs as a result of a medication’s low solubility in melting lipids (95). Furthermore, the high melting temperature of lipids may have an effect on the chemical stability of medications.

### 4.4 Microfluidic approaches

When fluids are controlled in microchannels with dimensions on the order of tens of microns, nanoprecipitation can create various nanoparticles (96). Unlike bulk nanoprecipitation, microfluidic techniques for creating LNPs provide several extremely desirable features, including reduced particle size, narrow size distribution, viability for scale-up, enhanced EE, and excellent repeatability (79). Microfluidic devices can generally be classified into two types for producing LBNPs: (1) microfluidic devices based on chips, and (2) microfluidic devices based on capillaries. Based on a design that uses hydrodynamic flow focusing (HFF). HFF uses two vertical shearing pressures to squeeze the central channel and create an extremely narrow focused stream, resulting in quick diffusion-based mixing (79). A comparison study was done for siRNA-LNPs made with an HFF microfluidic chip and vortex mixing (97). The microfluidic approach produced siRNA-LNPs with an average size of 38 nm, resulting in nanoparticles with a tighter size distribution and a 20% improvement in EE compared to those created using vortex mixing (98). Different micromixer structures were created to increase mixing efficiency and further improve the mixing caused by the diffusion force in HFF (99). The Tesla-structured HFF microfluidic device’s convoluted microchannels allow the fluid to be regularly divided and merged for rapid mixing (79). Lipid-polymeric hybrid nanoparticles were synthesized using the Tesla-structured HFF microfluidic device. In the fourth mixing cycle, the mixer could finish at a flow velocity of 10 m/s for 50 L. A range of nanoparticles with a well-controlled size distribution were produced using this technique, including polymer nanoparticles between 40 nm and 50 nm, lipid nanoparticles (LNPs) of around 250 nm, and polymer nanoparticles with a 40-nm lipid covering (100). The fundamental disadvantage of microfluidic HFF is the resultant nanoparticles’ relatively low concentration, which may require additional processes to concentrate (100).

### 4.5 Coacervation method

The coacervation approach, a new and solvent-free technology, was initially published for the synthesis of LBNPs by Battaglia et al. in (101) to eliminate the drawbacks connected with those methods stated above, such as hazardous organic solvents and sophisticated equipment (101). As pH decreases, a micellular solution of fatty acid alkaline salts precipitates due to the proton exchange caused by acidification between the acid solution and alkaline salts (101). Fatty acid LBNPs can be produced by progressively adding a coacervating solution that lowers pH to a certain threshold. Before adding the coacervating solution, the combination of equally dispersed lipids and surfactants should also be heated above the Krafft point of the fatty acid salt and often stirred to create a transparent solution (102). The coacervation process was used to create an ideal formulation of baicalin-loaded SLN with 0.69% w/v lipid and 26.64% w/w drug/lipid ratio. The synthesized SLN has a PDI of 0.169, an EE of 88.29%, and a particle size of 347.3 nm. The stearate sodium and 1% hydroxypropylmethylcellulose (HPMC) aqueous solution was agitated and heated over the stearate sodium Krafft point (47.5°C) until a clear solution was noticed. Afterward, baicalin was added as a model drug when the solution’s temperature was raised to about 60°C and all its components had dissolved. Drop by drop, HCl solution was added until the pH reached 4.0. After that, the solution was quickly cooled to 15°C and stirred in an ice water bath to create the final nanoparticle. To verify that the medication included in the nanoparticles was crystalline and that spherical nanoparticles had been produced, differential scanning calorimetry (DSC) was employed (103). This approach is straightforward but can only be used with lipids that can produce alkaline salts, such as fatty acids.

### 4.6 Supercritical fluid technology

The synthesis of nanoparticles using supercritical fluid (SCF) technology is a promising process that offers benefits like enhanced nanoparticle size control, uniform size distribution, complete solvent elimination, and environmental friendliness (104). By adjusting pressure and temperature, a substance with a supercritical form and tunable solvent power is used in SCF technology (105). Carbon dioxide is the most commonly utilized SCF because of its outstanding safety and affordable price. The most common method for producing LBNPs with SCF involves altering the ambient pressure of supercritical CO_2_ (scCO_2_) (106). In a nutshell, scCO_2_ is utilized as a solvent, and when fed into a high-pressure vessel, the solubilities of solid lipids and medications in scCO_2_ are boosted. The solid lipids and pharmaceuticals are then abruptly supersaturated and precipitate out, creating drug-loaded LNPs due to the depressurization process (79). Particles from gas-saturated solutions (PGSS), gas antisolvent (GAS), and supercritical antisolvent (SAS) are some of the various methods for the manufacture of SLNs that have been developed based on this SCF approach (107).

## 5 Drug resistance and epithelial mesenchyme transition

Drug resistance is a basic idea that limits the viability and utilization of sickness (108). A qualification ought to be set up between two kinds of drug resistance: acquired or multiple drug resistance (MDR), which occurs when a therapy generates protection from another combination of treatments (109). Essential drug resistance (DR) develops before starting any therapy. Proteins linked to drug digestion (such as cytochrome P450 and glutathione *S*-transferees) and film carriers that further modify confluence and drug efflux are likely the most essential tools of resistance to cancer (110).

The ability to use nanoplatforms to disrupt resistance mechanisms is a potential treatment technique for cancer (111). A folate-formed solid lipid nanoparticle can do this differentially and sequentially by providing two coexemplified anticancer drugs, paclitaxel (PTX) and curcumin (CUR), so that CUR, which is capable of inhibiting P-gp, begins to be administered sooner than PTX (112). As a result, P-gp inhibition in MCF-7/ADR BreC-safe cells is ensured, allowing PTX accumulation within. Likewise, different components have been engaged with multiple drug resistances, including DNA harm fix (expanded fix), epigenetics (microRNAs, histone alteration, DNA methylation), long noncoding RNAs, and oncogenes (KRAS, MALT1, p53, AKT, ERBB2, PIK3CA, HGAL) (113). The blended hyaluronic acid-corrosive/DOTAP liposome center/shell-NPs were layered with polymetformin, a dicyandiamide and polyethylenimine compound suitable for retaining vascular endothelial growth factor (VEGF) siRNA (114). In lung cancer (LuC)-bearing mice, this framework reduced VEGF by 95% while increasing tumor mass apoptosis (up to 40%). Be that as it may, the adequacy of this nanoplatform did not come uniquely from VEGF siRNA (115). The studies conducted without siRNA showed an antitumor response mediated by polymetformin, which inhibited mTOR and also started the AMPK channels responsible for the DNA damage response and the tumor silencer migration, respectively.

Finally, the epithelial-mesenchyme transition (EMT), which happens when epithelial cells stop communicating regular signs of separation (mostly E-cadherin) and start to communicate markers of mesenchyme cell separation (N-cadherin), may be necessary in the battle against malignant growth (116). This change has been connected to measures, for example, malignancy metastasis. Thus, it is hypothesized that EMT might promote cancer stem cell (CSC) characteristics by increasing MDR wonders. Such designed LBNPs that were functionalized with the RGD peptide and stacked with diacidic norcantharidin could accomplish authoritative binding to the phone layer protein-integrin 5 and restore the declaration of E-cadherin.

## 6 Tumor targeting of LBNPs

As standard chemotherapeutic agents only exhibit a small amount of tumor specificity and influence both ordinary and, moreover, tumor cells in a specific way (117), the successful portion required for the therapy of malignancy is not ideal as a result of the concurrent toxicity (118). The tumor focusing on the capacity of nanoparticle drug conveyance frameworks, counting lipid-based nanoparticles, for example, liposomes, points to an effective method to increase anticancer drug specificity for tumor cells while reducing toxicity to their usual companions (119). By explicitly focusing on tumor cells, nanoparticles can enhance the pharmacodynamics and pharmacokinetics characteristics of the drug, manage and maintain drug delivery, increase the drug’s specificity for tumor cells, enhance drug concealment and intracellular delivery, and lessen the drug’s overall danger (120). Two different approaches to treating tumors, active and passive targeting, are described below (121).

### 6.1 Passive targeting

**Figure 4:**
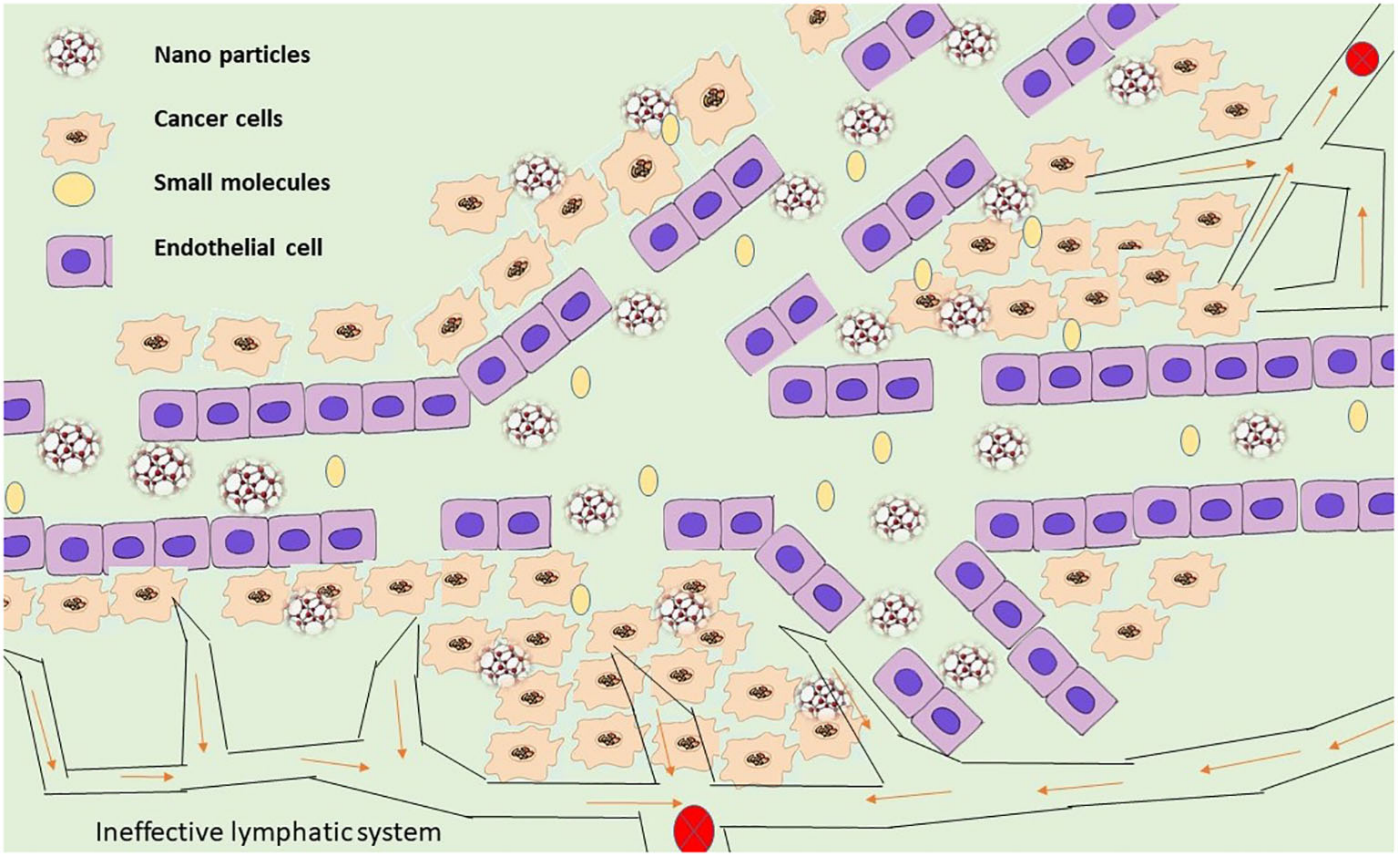
Cancer-targeting nanoparticles that operate passively.

The characteristics of the delivery mechanism and the disease life systems are used in passive targeting to explicitly aggregate the medication at a focused availability and evade vague dissemination (122). In comparison to ordinary blood vessels, tumor veins are unique from multiple points of view. They are mostly described by variations from the norm, for example, high extents of multiplying endothelial cells, predicate insufficiency, and abnormal storm cellar film arrangement (123). Most tumor capillary arteries exhibit enhanced capillary permeability and are recognized to be damaged due to these anomalies (124). Given that most fringe tumors have defective veins made of permeable endothelium covering with hole sizes estimated to be between 400 nm and 600 nm, it is acknowledged that particles as small as 10 nm to 500 nm can extravagate and aggregate inside the tumor interstitial space (**Figure 4**).

Aside from improved vascular porousness, tumors also show a nonfunctional lymphatic framework, which adds to useless waste inside tumor tissues (125). Nanoparticle aggregation occurs inside the tumor due to the lymphatic system’s inability to effectively remove any nanoparticles that enter tumors (126). The impact of EPR is a latent phenomenon that plays a crucial role in the latent focus of drugs and nanoparticles (127). The attributes of tumors and tumor veins, for example, (1) broad angiogenesis (128); (2) hypervasculature (129); (3) conflicting and violent bloodstream (130); and (4) slow venous return (54) that prompts molecule gathering inside the tumor interstitial, additionally help add to the enhanced permeability and retention impact of detached focusing. It has been suggested that due to the tumor’s design, the extracellular liquid weight must be considered (131). It is presently perceived that most strong tumors show an expanded interstitial liquid weight. Although vascular changes may not facilitate the movement of nanoparticles through tumoral veins into the tumor interstitium, an increased tumor interstitial liquid weight may prevent the effective transcapillary growth of nanoparticles into tumors (132). To precisely collect the encapsulated material at a particular place, passive targeting takes advantage of the delivery mechanism’s characteristics and the illness’s anatomy (133). The EPR effect is a mechanism that underlies nanoparticles’ passive targeting of malignancies (124).

The control of nanoparticle size and surface charge, just as the expansion of PEG or polyethylene oxide, can additionally assist with improving the viability of aloof focusing (134). It is recognized that nanoparticles less than 200 nm wide and those with a modest positive charge tend to concentrate inside tumors for a longer time than neutral or negatively charged nanoparticles (135). The surface modification of nanoparticles with either PEG or polyethylene oxide also takes into account a longer nanoparticle dispersion time by reducing opsonic bond and opsonization, which consequently reduces nanoparticle recognition by RES (136). Finally, this increases the aggregation of nanoparticles in solid tumors, as demonstrated by the covert PEG-related liposomes loaded with doxorubicin (Doxil) and poly(ethylene oxide)-altered poly(caprolactone) nanoparticles containing tamoxifen (137). Dynamic focusing on the cycle may then occur after uninvolved focusing is complete, more specifically after the conveyance framework has accumulated inactively inside the tumor site.

### 6.2 Active targeting

**Figure 5:**
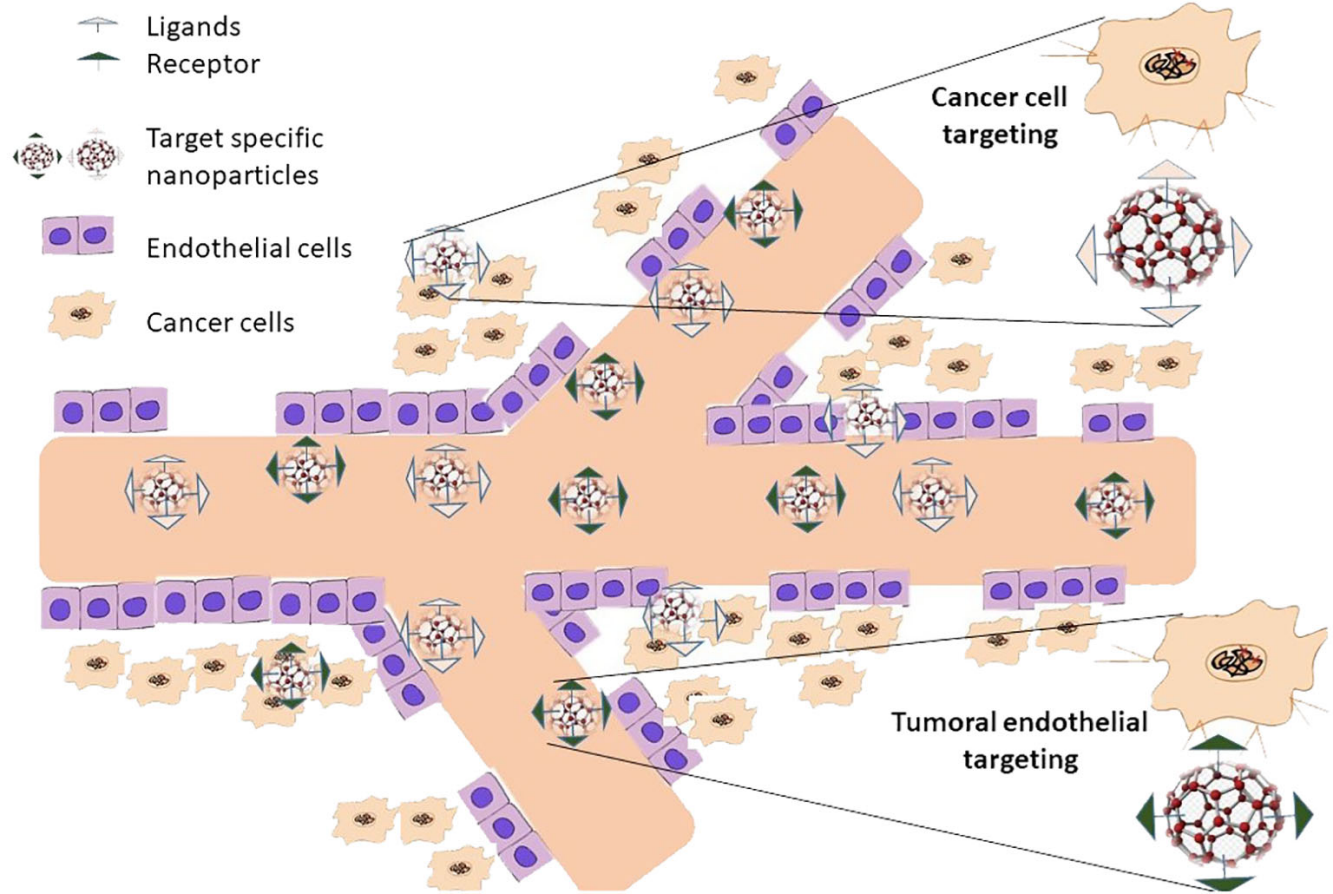
Nanoparticles actively target cancer tumors with their attacks. In active targeting, unique ligands attached to nanoparticle surfaces that are selectively recognized by cells at the disease site enable the nanoparticles to interact solely with these cells. Only once passive targeting is finished can active targeting take place. This suggests that it can only happen after passive nanoparticle accumulation at the illness location.

In addition to passive targeting, active targeting techniques may be used to tailor nanoparticle drug delivery frameworks to be more selective to cancer cells (138). In active targeting, specific ligands that are detected by disease-site cells are attached to the exterior of nanoparticles, enabling them to interact with tumor cells directly (139) (**Figure 5**). The most well-known technique for active targeting involves employing a ternary complex composed of an active medication, a ligand or immunological reaction as a focal moiety, and lipids or polymers as a carrier (140). While planning ternary structures, a few factors that focus on moiety must be considered to produce a viable conveyance framework. To begin with, a cell receptor and its ligand must have characteristics that qualify them as tumor-explicit targets (141). For instance, the receptor should be produced excessively in disease cells, not normal cells. Second, the picked receptor ought to be communicated homogeneously outside of all directed disease cells (142). Third, the ligand–receptor complex should not be delivered into the blood circulation after the ligand binds to its receptor (143). After formal approval, the ligand–receptor combination must finally be hidden inside the directed cell.

The early endosome’s increased causticity can affect the compliance of several receptors and, in this way, permits related ligands to be formed (144). Depending on how well the receptor/ligand combination works, the receptor and its ligand will either return to the plasma film or continue down the end lysosomal route for lysosome debasement in a late endosome (145). For instance, it is acceptable to reuse some receptors in the plasma, such as the transferrin receptor and its ligand, low-density lipoprotein receptors, and folate receptors (146). While low density lipoprotein (LDL), α-2- macroglobulin and the epidermal growth factor receptor (EGFR) and its ligand are transferred to multivesicular bodies (MVBs) which then developed into late endosomes for lysosomal degradation (147). It is crucial to remember that the precise mechanism by which various endocytoses occur is currently unclear and needs more research. Nanoparticles can be directed to tumoral endothelium or diseased cells when active targeting is used (148).

## 7 LBNPs with drug encapsulation

### 7.1 Water-soluble drugs

Due to their high water solubility, hydrophilic medicines are difficult to encapsulate and control their release precisely, making it challenging to prevent drug leakage into the aqueous phase. LBNPs have been considered a viable means of trapping and administering hydrophilic medications. Hydrophilic drug encapsulation has made extensive use of microemulsions and double emulsions. A microemulsion method was created to encapsulate promethomycin, a broad-spectrum antibiotic used to treat parasite infections. This chemical compound is very polar and has an extremely high water solubility of 79.9 mg mL^−1^. A maximum EE (41.65%) of paromomycin in stearic acid nanoparticles was obtained using the microemulsion process, with a drug-to-lipid ratio of 4. A clear stearic acid and surfactant combination was stirred while an aqueous phase containing paromomycin at 85°C was introduced. To create nanoemulsions, the generated microemulsion was homogenized for 20 min at 18,000 rpm. After cooling in double-distilled water (2°C–4°C), stearic acid nanoparticles containing paromomycin were then produced. Hydrophobic medications are also frequently encapsulated using double emulsions. W/O/W double emulsions’ distinct structure and characteristics allow for the creation of hydrophilic drug-loaded LBNPs.

### 7.2 Water-insoluble drugs

Since hydrophobic medications comprise 90% of currently being developed pharmaceuticals and 40% of authorized pharmaceuticals, there has been a lot of interest in creating delivery systems for these treatments. For the delivery of hydrophobic drugs, LBNPs have been widely utilized. Numerous hydrophobic medications have been effectively encapsulated in LBNPs, enhancing bioavailability and regulated release. One such powerful antiangiogenic and antineoplastic drug is docetaxel (DTX). The LBNPs obtained 86% EE of DTX, 2% DL, and a regulated release profile. Their particle size was 128 nm with a PDI of 0.2. The final DTX-loaded LNPs were noteworthy for their 120-day stability. Similar to this, Das et al. used an emulsification-ultrasonication approach to generate tretinoin-loaded LNPs with > 75% EE utilizing Proprietrol ATO5 and Compritol 888 ATO. For 3 months at 4°C, the LBNPs infused with tretinoin remained stable. Furthermore, utilizing a solvent-diffusion technique, IR-780 iodide-loaded c(RGDyK)-conjugated LNPs were created for near-infrared (NIR) imaging-guided photothermal therapy. In mouse tests, a high EE (85.34%) was attained together with significant cytotoxicity and few side effects. Moreover, LBNPs have been created to encapsulate medications like cisplatin (CDDP), which is soluble neither in water nor in oil. Such medications’ poor solubility presents difficulties for drug delivery system research and design. Gupta et al. achieved a high EE of up to 80.8% in the effective synthesis of lipid-coated CDDP nanoparticles.

## 8 Applications of LBNPs in cancer therapy

A sizable and varied collection of nanoparticles called LBNPs is crucial for treating BreC (149). Despite this diversity, however, liposomes are commonly used because they are extremely biocompatible and capable of encapsulating a wide variety of loads (150). LBNPs are commonly used in many studies, and some have already been approved for BreC treatment (Doxil^®^ or Abraxane^®^) (149). The most significant advancements in LBNP usage for the treatment of the most prevalent cancer types are covered in this section. Clinical trial status of different LBNPs for multiple cancers have been exhibited in **Table 2**.

**Table 2:** Clinical trial status of lipid-based nanoparticles in drug delivery in multiple cancers.

Drug/NP/intervention	NCT	Cancer type	Status	Phase
Mitoxantrone hydrochloride liposome injection	NCT04719065	Advanced solid tumor	Active, not recruiting	Phase I
Fluorouracil irinotecan liposome	NCT03837977	Neuroendocrine carcinoma	Active, not recruiting	Phase II
Irinotecan liposome injection	NCT03088813	Small cell lung cancer	Active, not recruiting	Phase III
PEGylated liposomal doxorubicin	NCT05830539	Locally advanced or metastatic solidctumor	Active, not recruiting	Phase II
Liposomal doxorubicin	NCT05354076	Advanced malignant tumors	Recruiting	Phase II
Liposome doxorubicin	NCT05561036	Desmoid tumor	Recruiting	Phase III
Mitoxantrone hydrochloride liposome	NCT05620862	Lymphoma and solid tumors	Recruiting	Phase I
FF-10850 topotecan liposome injection	NCT04047251	Solid tumors	Recruiting	Phase I
FF-10832 gemcitabine liposome injection	NCT03440450	Solid tumors	Recruiting	Phase I
188Re-BMEDA-liposome	NCT02271516	Primary solid tumor	Terminated	Phase I
Liposomal doxorubicin	NCT00819221	Solid tumor	Terminated	Phase I
Liposomal bupivacaine	NCT03867188	Sarcomas	Terminated	Early phase 1

### 8.1 Gastric and esophageal cancer

Gastric cancer (GC) is the third leading form of cancer-related mortality worldwide and the fifth most prevalent cancer overall (151). Gastric cancer may be managed solely with surgical excision without lymph node metastases. However, established stomach cancer requires combination chemotherapy, which has serious side effects (152). New therapies are currently being developed based on the use of nanoformulations to enhance patient response. In GC therapy, liposomes have frequently been employed in conjunction with compounds like the Arg-Gly-Asp peptide SATB1 siRNA, CD44 antibodies, or DNA complexes (149). Their usage has enhanced the accumulation of medicines in tumor-bearing mice that received SGC7901 cells with elevated integrin expression as a transplant. Additionally, liposomes showed better targeting precision and had a % silencing effect on SATB1 gene expression in CD44+ GC starting cells (149, 153).

Additionally, liposome diseases were able to identify peritoneally disseminated GC MKN-45P cells, reducing liver accumulation. Preliminary tests of SLNs in GC showed increased topside activity in SGC-7901, increased inhibition of development, a cell arrest in the G2/M process (17.13%), and the activation of mitochondria-dependent apoptosis, respectively (57). On the other side, several novel NPs for esophageal cancer (EC), the seventh most prevalent malignancy globally, have been published (131). It is used with radiotherapy the rhenium-188 (188Re)-liposome to test its efficacy in tumor-bearing mice BE-3 (esophageal adenocarcinoma). On the other hand, the effectiveness of radiation combined with the well-known rhenium-188 (188Re)-liposome in treating tumor-bearing mice BE-3 was examined in the case of esophageal cancer (EC), the sixth most common cancer in some recent NPs globally (esophageal adenocarcinoma) (57).

### 8.2 Pancreatic cancer

Early identification of pancreatic cancer (PC) has not been carried out with the screening. As a result, when an operation cannot be done, PaC is always noticed in the late stages (154). However, the ineffectiveness of pancreatic cancer treatments is due to both the tumor’s late stage (often with metastases) and the medications’ inability to penetrate deeply due to the presence of tumor stomas (155). Nanotechnology offers specific therapeutic ways to enhance certain patients’ prognoses. The steroid was prepared using ME cold dilution techniques for chitosan-coated lipid nanoparticles and filled with CUR (156). This mechanism has increased cell growth inhibition (threefold) in PANC-1 cells for CUR (10 μM). The tocotrienol isomer of vitamin E and the T3-T-mPEG 2000 core/corona nanoemulsion have both been shown to increase the effectiveness of gemcitabine (the first-line therapy for PC) (NE) (157).

In comparison to free gemcitabine, this relationship showed that Bx-PC-3 cells and gemcitabine-resistant PANC-1 cells had stronger anticancer activity (158). Additionally, tests with gemcitabine utilizing MIAPaCa xenograft designs revealed human plasma albumin complexes linked to PTX and thermosensitive liposomes, resulting in allergic acid (159). PEG-EF24 (a synthetic CUR analog)-liposomes hindered the capacity to form colonies of MIAPaCa and Pa03C cells *in vitro* and demonstrated synergistic tumor development inhibition when assessed *in vivo* (160). The system improved PTX cellular absorption and half-life while reducing tumor growth in animals with BxPC-3/HPaSteC adhesion when combined with heat (161). Finally, several of the newly found medicinal methods have undergone human testing. In this area, it is important to note a phase III clinical trial examining the use of nanoliposomal IRI (nal-IRI) to cure advanced PaC (162).

### 8.3 Liver cancer

The treatments for lung cancer (LC) are frequently constrained by the drug’s subpar physicochemical characteristics (163). Indeed, chemotherapy and selective medicines such as sorafenib have limited implications for the survival of patients. Moreover, radiation treatment is normally ineffectual because it can unintentionally affect neighboring healthy tissues, resulting in side effects and problems; also, its effectiveness decreases with metastatic malignancies. (93). Combining medications with certain nanoplatforms has been suggested as a technique to improve the overall effectiveness of the medication and patient survival (164). In this way, PTX and 5-FU-charged NLCs were utilized in the management of male patients with LivC. On the one hand, 5-FU-charged autosomes quickly shield the medication from enzymatic deterioration by boosting its hepatic accumulation (165). In addition to the Intaxel^®^ commercial formulation results, the PTX-laden NLCs benefit from its buildup and permanence in plasma. To achieve the dual treatment for HepG2 cells (166), a device containing sorafenib and SPIONs coloaded in SLNs has also been created.

### 8.4 Nervous system cancer

The most severe and widespread type of malignant brain tumor (50% of cases), glioblastoma multiform (GBM), has a 5-year surveillance rate (10%), which is extremely low and has a 10% survival rate (167). Temozolomide (TMZ) and radiation, as used in surgical resections, still suggest modest survival rates (15 months) (168). Resections from the whole tumor, healthy brain tissue infiltrations, and the blood–brain barrier, which inhibits medication distribution from the bloodstream to the brain, are all downsides of current therapy. The outcomes of the treatment are limited. Myocet^®^, a non-PEG-DOX liposome, has been utilized in individuals who received TMZ-based chemotherapeutic therapy (phase I clinical trial) in high-grade glioma (oligodendroglia or oligoastrocytoma, astrocytoma, GBM) (57). To determine the maximum tolerable dose, people (aged 28 to 65) with high-grade gliomas received nanoliposomal IRI (nal-IRI) (169).

### 8.5 Lung cancer

Despite effective chemotherapy and radiation therapies for lung cancer, LuC is one of the most prevalent malignancies and the leading cause of cancer-related mortality in both men and women globally. Most patients are sadly more resistant to subsequent therapies and have a recurrence of the disease (170). Therefore, a novel treatment approach is required to enhance the prognosis for this class of malignancies. The signification of and application of nanotechnologies to diagnose and treatment of this form of cancer has been substantially advanced in recent years, using LBNPs as well. For instance, it has been demonstrated that docetaxel-NE and NE lipophilic diffuse methane have improved antitumor action in cells of A-549 (171). Even more successful than normal cells was nebulized docetaxel NE (MRC-5 cells) against tumor cells (A-549 cells), creating a standard framework for coencapsulating gemcitabine (GEM) and PTX with a ligand that targets the glucose receptor (172). These researchers demonstrated that a 3:1 ratio of GEM and PTX in A-549 resulted in a potent synergism. Finally, some clinical studies revealed successful results for lung cancer. For instance, a phase III clinical study included PTX intratemporal liposome injection (173).

### 8.6 Breast cancer

The incidence of cancer-related deaths in women has significantly risen recently (174), due to the treatment of advanced cannery stages and the advancement of LBNPs. When NEs were loaded with DOX and bromotetratrandrine (W198, a P-glycoprotein (P-gp) inhibitor), it was tested on the resistant MCF-7/ADR cell line (57). The tumor tissue has become more consumed by the cell and accumulated by DOX. Interestingly, DOX has demonstrated reduced gastrointestinal and heart toxicity (175). Additionally, DOX-liposome-based formulations were tested in clinical trials. PEG-DOX liposomes (PLD) in combination with lapatinib have recently been found as the most effective combination of both therapies with the maximum dosage tolerance in HER2-positive BreC individuals (phase Ib) (57). Xenograft-bearing mice might enhance the amount of DOX in tumor tissues by using an NLC coloaded with DOX and apache instead of free DOX in MCF-7/ADR tumors (176).

### 8.7 Prostate cancer

The three main LBNPs that are now being investigated as prostate cancer (PrC) therapeutic alternatives are NEs, liposomes, and SLNs (149). The NE oil in water contains an omega-3 fatty acid-related taxoid medication. Compared to Abraxane™, this NE could lower the toxoid IC_50_ of PPT2 cells by 12 times, leading to a larger decrease in tumor volume in tumor-bearing mice (149). The use of Catwechin-extract NE in PC-3 cells (flavones with anticancer properties) also shows similar antitumor advantages. With regard to liposomes, the PEG-folate-targeting oleuropein liposome was introduced to 22Rv1 PrC cells. Oleuropein bioavailability, 22Rv1 cell apoptosis, and *in vivo* survival have all been enhanced by these nanoplatforms. By integrating the device with the use of a laser, NPs made of multifunctional liposomes loaded with docetaxel and gold nanorods were created, which demonstrated a 100% inhibition of PrC cell growth (177).

## 9 Conclusion

LBNPs have been extensively employed in preclinical research as well as clinical settings for drug delivery applications. Numerous LBNPs have received approval for use in clinical settings, proving their distinct benefits over alternative drug delivery methods. In this review, three lipid-based drug delivery systems with LBNPs have been highlighted. Different methods and techniques are discussed for the synthesis of LBNPs. A wide range of water-soluble and water-insoluble drugs have been successfully explained. Applications of LBNPs against different types of cancer have also been discussed. Developing novel lipids and enhanced LBNP formulations will open up new possibilities for their use in drug delivery applications. One further major obstacle is the distribution of drugs with specificity. Even though ongoing attempts are being made to create novel targeted delivery methods, clinical reality is still far off. Several techniques that show promise have been explored for targeted delivery of LBNPs. Lipid components also affect how medications are biodistributed. Whereas DSPC-formulated LBNPs favor the spleen, DOPE-formulated LBNPs tend to collect in the liver. Future designs of more efficient LBNPs for drug administration will undoubtedly benefit from a deeper comprehension of the structure–function interactions and lipid chemistry. Additionally, cutting-edge technologies like machine learning and meta-data analysis in published research will offer substantial resources for LBNP design in the future.

## Author contributions

IW and AA: Conceptualization, Formal analysis, Methodology, Software, Writing – original draft, Writing – review & editing. HT: Investigation, Visualization, Writing – review & editing. NK: Conceptualization, Validation, Writing – review & editing. AA: Conceptualization, Supervision, Writing – original draft, Writing – review & editing. XZ and W-FL: Funding acquisition, Project administration, Resources, Supervision, Validation, Writing – original draft, Writing – review & editing.
